# Alcohol-related hypoglycemia in rural Uganda: socioeconomic and physiologic contrasts

**DOI:** 10.1186/1865-1380-4-5

**Published:** 2011-02-10

**Authors:** Heather Hammerstedt, Stacey L Chamberlain, Sara W Nelson, Mark C Bisanzo

**Affiliations:** 1 Idaho Emergency Physicians, Boise Idaho, USA; 2 Department of Emergency Medicine, University of Illinois at Chicago, Chicago Illinois, USA; 3 Department of Emergency Medicine, Maine Medical Center, Portland Maine, USA; 4 Department of Traumatology and Emergency Medicine, University of Connecticut, Hartford Connecticut, USA; 5 Global Emergency Care Collaborative

## Abstract

Hypoglycemia is a rare but important complication seen in patients who present with alcohol intoxication. In a study by Marks and Teale, less than one percent of people with alcohol intoxication who presented to an American emergency department were hypoglycemic [1]. It is even more rare to see an intoxicated patient, who had been eating appropriately prior to or during the intoxication, present in a hypoglycemic coma. However, our analysis of the first 500 patients seen in a newly opened five-bed Emergency Department (ED) at Nyakibale Karoli Lwanga Hospital in rural southwestern Uganda, revealed multiple intoxicated patients who presented in hypoglycemic coma within hours of eating a full meal. Three of these cases are summarized and discussed below.

## Case One

A 32-year-old man was found confused and moaning in bed by family at 5 a.m., and brought in by family at 9 a.m. Family members stated he had eaten lunch and dinner with them the previous day, then went out drinking alcohol with friends and came home at 3 a.m. Past medical and surgical histories were unremarkable, and he takes no medications and has no allergies.

On examination, his vital signs were stable (blood pressure 110/70 mmHg, heart rate 68 bpm, respiratory rate 12 bpm, oxygen saturation 93% room air, temperature 37°C), and the patient was unresponsive. He responded to sternal rub with moaning and moved all his extremities to painful stimuli. He smelled of sweet alcohol and did not answer questions. His eyes were open, pupils were reactive, and his head was normocephalic and atraumatic. He had no meningismus and no clonus. Cardiopulmonary and gastrointestinal examinations were normal, and he had no signs of trauma. A fingerstick point of care test indicated that the concentration of glucose in his blood was 27 mg/dl.

The patient was given 30 ml of D50W, awoke immediately, jovial and smiling, and was observed for 1 h. While getting 500 ml of D5W, he ate some food, remained normoglycemic, and then was discharged. He did not return within 1 month. His diagnosis was alcohol-related hypoglycemia.

## Case Two

A 50-year-old man, known to be an alcoholic, presented after being found unresponsive at home in bed. He had been drinking the night before, but his family members could not arouse him in the morning. He had eaten all three meals the day and night before. Further history elucidated that he had had a cough for 1 month and 2 days of epigastric pain without vomiting, hematochezia, or diarrhea. He had no remarkable medical or surgical history, took no medications, and had no known drug allergies.

His examination demonstrated a disheveled man who appeared unresponsive with only gurgling respirations (temperature 34.3°C, pulse 96, blood pressure 90/50, respiratory rate 20, oxygen saturation, 86% room air). He had no signs of trauma. His pupils were reactive and equal. He moved all extremities to painful stimuli and sternal rub, and his cardiopulmonary examination was normal. He had no meningismus and no clonus. His abdominal examination revealed epigastric guarding. There was no gross blood on rectal examination. His blood glucose concentration was 19.8 mg/dl as determined by a fingerstick. A chest x-ray was obtained because of hypoxia and demonstrated a possible left lower lobe infiltrate.

The patient was given 25 ml of D50W and 500 ml of D5W. He awoke rapidly, and the results of his neurologic examination were normal, but he remained hypothermic and hypoxic with epigastric guarding. He was given oral omeprazole (as only oral proton pump inhibitors are available in Uganda), IV ranitidine, and IV ceftriaxone, and placed on an oxygen concentrator. The next day, the patient improved and was normothermic and normotensive, with normal oxygen saturation, and with normal abdominal and cardiopulmonary examination results. The patient was discharged with the diagnosis of alcohol-related hypoglycemia, gastritis, and aspiration pneumonia. He declined prescriptions for proton pump inhibitors or antibiotics on discharge. He did not return within 1 month.

## Case Three

A 55-year-old female presented to the ED after being found unresponsive in bed by friends in the morning. She was last seen by her neighbors the night before when they ate dinner together. Her neighbor stated that the patient does not regularly consume alcohol. Her past medical history was significant only for peptic ulcer disease, but she was not currently taking any medications. She had no known drug allergies.

Examination revealed a well-nourished, hemodynamically stable (temperature 37°C, pulse 113, blood pressure 152/98, oxygen saturation 95% room air), non-toxic, unresponsive female. She withdrew all extremities to painful stimuli, and had no clonus and no meningismus. She had no signs of traumatic injuries; her pupils were reactive, and her cardiopulmonary and gastrointestinal examinations were normal. The blood glucose concentration, as determined by fingerstick, was 32 mg/dl.

The patient received 15 ml of D50, improved immediately to a normal mental status, and had normal neurologic examination results. The patient reported that she had been drinking large quantities of alcohol, but she had eaten all her meals yesterday. The patient was admitted to the ward. She continued to be alert and oriented, and was discharged the next day. She did not return within 1 month. Her diagnosis was alcohol-related hypoglycemia.

## Discussion

In the United States, alcoholic patients who present with a depressed mental status are usually not in a hypoglycemic coma, but instead have other etiologies of coma such as sepsis, shock, hypothermia, trauma, or excessive intoxication. Those who do present in a hypoglycemic coma usually are an exception to this 'rule' and fit into a few clinical stereotypes. Most have had a prolonged calorie fast (greater than 24 h) in the setting of an extended alcoholic binge or have a fasting state of several days with a more moderate amount of alcohol ingestion [2-4]. Additionally, these patients are usually abstaining from alcohol by the time of presentation [5]. Also, in the US, it is rare for non-diabetic patients to present with hypoglycemia; in one study over an 8-year period, only 88 patients were admitted to a tertiary care hospital with hypoglycemia, and of these, alcohol intoxication was found in only 13 patients (15%) [6]. Our Ugandan patients seem to differ from US patients in that it was common to observe non-diabetic patients presenting with hypoglycemia; also, the Ugandan alcoholic patients did not fit into any of the 'exceptions' noted above as there was no fasting state, and they presented after acute alcohol ingestions without a significant period of abstinence.

To understand this, it is necessary to review the basic pathophysiology of glucose utilization [7]. In the postprandial state, insulin levels peak at about 1 h and then steadily fall over the next several hours. Simultaneously during this decline, there is decreased uptake of glucose by the liver, muscle, and adipose tissue. Insulin is no longer suppressing glycogenolysis, gluconeogenesis, and lipolysis. For the first 12-24 h of fasting, hepatic glycogenolysis provides most of the glucose from glycogen stores. After that, lipolysis and protein breakdown provide fatty acid for energy, and glycerol and amino acids for gluconeogenesis. Therefore, assuming that a person is eating adequately at regular intervals, hypoglycemia can be avoided through various back-up mechanisms.

Alcohol affects this process however. When ethanol is metabolized in the liver by alcohol dehydrogenase to acetaldehyde, it reduces nicotinamide adenine dinucleotide (NAD) to NADH. In the next step in the pathway of ethanol breakdown, acetaldehyde is metabolized by aldehyde dehydrogenase, which also reduces NAD to NADH and produces acetate, which leaves the liver for metabolism by extra-hepatic tissue, such as skeletal muscle. Thus, the process of alcohol metabolism significantly decreases the hepatic NAD/NADH ratio. In rats, where the effect of ethanol on the hepatic redox state has been determined by freeze-clamping the liver, this ratio changes rapidly from 700/1 to 200/1 [8]. This change in hepatic redox state has profound effects on metabolic processes in the liver, since both alcohol metabolism and gluconeogenesis occur in this tissue, and both metabolic processes alter the NAD/NADH ratio.

Gluconeogenesis requires a specific NAD/NADH ratio for the reductive synthesis of glucose [9]. Elevated levels of NADH, such as occur during ethanol metabolism, negatively affect a number of critical dehydrogenases in the liver that are required for gluconeogenesis [10]. For example, the conversion of lactate to pyruvate (a key gluconeogenic step) will be strongly inhibited by the increased level of NADH caused by the oxidation of ethanol. In addition, malate conversion to oxalacetate by NAD malate dehydrogenase, which is a critical reaction in gluconeogenesis, will be inhibited by the high level of cytosolic NADH. Alanine is another key gluconeogenic intermediate whose metabolism is profoundly altered by ethanol oxidation. Alanine is converted to pyruvate in the liver by alanine aminotransferase, but the elevated levels of NADH ensure that pyruvate will be immediately converted to lactate by lactate dehydrogenase. Thus, ethanol consumption rapidly increases the blood lactate concentration, while decreasing the level of glucose. Ethanol also can redistribute pancreatic microcirculation to enhance late-phase insulin secretion. This can cause hypoglycemia directly and inhibit the release and activation of counter-regulatory corticotropin, cortisol, and growth hormone that normally counteract hypoglycemia, which increases the risk for reactive hypoglycemia [11].

The hepatic redox state, however, should not affect the other processes of glycogenolysis and lipolysis, the other two major sources of glucose and energy other than gluconeogenesis. So why would our intoxicated Ugandan patients present with hypoglycemia despite normal feeding patterns when the US patients do not? Both lipolytic and ketogenic pathways are important to prevent hypoglycemia by providing substrates, free fatty acids, and ketone bodies, for alternative energy sources. If levels of fatty acids or ketone bodies fail to increase during fasting, the risk of hypoglycemia increases. The Ugandan diet is mostly carbohydrate starch from matoke (boiled mashed banana) and cassava, with a small amount of protein intake from beans and legumes. The impoverished low socioeconomic rural population that we serve in our hospital does not typically have access to a more varied diet. Patients commonly demonstrate kwashiorkor (protein deficiency) and marasmus (combined protein and carbohydrate deficiency) syndromes. It is possible that due to this chronic malnutrition, they lack the appropriate hepatic and muscle glycogen reserves that would be required to maintain normal blood glucose levels after even a brief fast combined with alcohol consumption [12].

Chronic malnutrition also decreases the availability of triglyceride in adipose tissue, which results in deficient glycerol available to be converted to glucose and fewer fatty acids to support energy metabolism. The average weight of an adult man in our ED is approximately 50 kg. These patients would then not only have gluconeogenesis limited by alcohol metabolism (as our US alcoholic population does as well), but they also have an inability to back up glucose stores with appropriate glycogenolysis and lipolysis. Despite only a short fast, in this baseline malnourished state, the normal metabolic mechanisms, such as mobilization of hepatic glycogen and fatty acid from adipose, would be insufficient to maintain energy homeostasis in the face of an ethanol-induced decrease in hepatic gluconeogenesis.

Many other factors could be involved in this process as well. Further research should be undertaken to investigate the possibility of a genetic difference in the rate of clearance of ethanol (which could maintain the reduced hepatic redox state longer than expected). Perhaps the type of alcohol (or its processing) could play a role as well. Most of the rural population in this area drinks homemade sorghum or banana alcohol instead of other commercial grain alcohols or beers seen in more socioeconomically sound communities in high income countries. A less likely theory would be a frank baseline NAD deficiency due to genetics or malnutrition, but one would expect a more global effect on health rather than just alcohol-induced hypoglycemic coma. Little information exists on alcohol-related hypoglycemia in low-income countries, although hypoglycemia was commonly found in one recent study of alcoholic Nigerians [13].

## Conclusion

Socioeconomic factors in low and middle income countries clearly affect clinical scenarios in many ways, from higher infectious disease prevalence and an increasing proportion of chronic disease, to the financial and cultural barriers to access of care. As demonstrated in this case series, it seems that socioeconomic factors may also play a large role in affecting the clinical presentations of patients because of their effect on basic science pathophysiology. Alcohol-induced hypoglycemia may be only one example of this phenomenon; further research should be done to elucidate how these factors can affect other basic pathophysiologic processes.

## Competing interests

The authors declare that they have no competing interests.

## Authors' contributions

HH and SC participated in clinical care of all case studies. HH was primary author. SC, SN, MB were secondary authors and editors. All authors read and approved the final manuscript.

## Consent

Verbal consent was obtained by the patients for publication of this case report, with 'written' thumb print as signature due to language barrier and high rates of illiteracy. These consents are at Karoli Lwanga Nyakibale Hospital in Rukungiri District, Uganda. Research approval was obtained by the Medical Superintendent of the hospital.
